# Analytical Imprecision and Reference Change Values for Longitudinal Monitoring of NCD-Related Biochemical Analytes

**DOI:** 10.3390/diagnostics16101532

**Published:** 2026-05-18

**Authors:** Siti Nurwani Ahmad Ridzuan, Muhammad Nursyazwan Zamre, Fadzlyasraf Shaari, Ahmad Asyraff Iqbal Anuar, Noor Hafizah Hassan, Nurul Izzati Hamzan

**Affiliations:** 1 Special Protein Unit, Specialized Diagnostic Centre, Institute for Medical Research, National Institutes of Health, Jalan Pahang, Kuala Lumpur 50588, Malaysia; siti.nurwani@moh.gov.my (S.N.A.R.); drnursyazwan@moh.gov.my (M.N.Z.); hafizahh@moh.gov.my (N.H.H.); 2 Alfa Diagnostik Sdn Bhd (Co. No. 1033482-X), No. 13, Jalan Delta U6/18, Taman Perindustrian Sunway Subang, Seksyen U6, Shah Alam 40150, Malaysia; fadzly@alfadiagnostik.com.my (F.S.); asyraffiqbal@alfadiagnostik.com.my (A.A.I.A.)

**Keywords:** analytical imprecision, internal quality control, non-communicable diseases, primary healthcare, reference change value

## Abstract

**Background:** Internal quality control (IQC) data offers continuous insight into analytical performance under routine conditions. This study evaluated IQC practices and long-term analytical imprecision (CV_a_) across primary healthcare laboratories to derive analyte-specific reference change values (RCVs) for non-communicable disease (NCD) monitoring. **Methods:** A 22-month retrospective analysis of IQC data was conducted across 29 primary healthcare laboratories using 32 analytical units (Beckman Coulter AU480) in Malaysian primary healthcare. Six analytes were assessed: glucose, creatinine, total cholesterol, triglycerides, HDL cholesterol, and ALT. CV_a_ was estimated using median and 90th percentile (P90) coefficients of variation across two concentration levels. RCVs were calculated at 95% probability (Z = 1.96) by integrating observed CV_a_ with within-subject biological variation (CV_i_) from EFLM databases. **Results:** IQC testing was highly standardized (median: 20 measurements/month). Long-term data showed stable, concentration-dependent imprecision. Median CV_a_ was lowest for glucose and lipids (1.7–1.9%) but higher for ALT (3.79%) and creatinine (3.52%) at Level 1. Derived RCV ranged from 14% (glucose) to 55.1% (triglycerides), with CV_i_ being the dominant contributor to RCV magnitude for most analytes. **Conclusions:** Long-term routine IQC data provide an analytically realistic foundation for deriving RCV in primary healthcare by reflecting real-world performance. Applying these RCV supports evidence-based interpretation of serial results, enhancing NCD monitoring by distinguishing true physiological change from analytical and biological noise.

## 1. Introduction

Internal quality control (IQC) is a cornerstone of laboratory quality systems and provides continuous assurance that analytical measurements remain stable and reliable under routine operating conditions [1]. Beyond its immediate role in error detection, IQC generates a rich body of longitudinal data that reflects real-world analytical performance over time. When systematically evaluated, these data offer valuable insight into long-term analytical imprecision as it is experienced in everyday clinical service rather than under controlled validation conditions [2].

Analytical imprecision, commonly expressed as the coefficient of variation (CV_a_), is an inherent component of all laboratory measurements and persists even when analytical systems meet manufacturer specifications and regulatory performance requirements [3]. Even when assays meet analytical performance specifications, residual imprecision persists and may influence the interpretation of small changes between consecutive results. For laboratories supporting longitudinal testing, particularly in high-throughput primary healthcare settings, an accurate understanding of CV_a_ under real-world conditions is essential to avoid overinterpretation of small numerical differences between consecutive results [4].

The reference change value (RCV) provides a quantitative framework to support longitudinal interpretation by defining the minimum difference between two consecutive measurements from the same individual that is likely to exceed expected analytical and within-subject biological variation [5]. By integrating observed CV_a_ with established within-subject biological variation (CV_i_), the RCV allows differentiation between true physiological change and normal variability [6]. The clinical usefulness of RCV, however, depends critically on the representativeness of the analytical imprecision estimates used in their derivation [7]. Precision estimates obtained from short-term validation studies may not adequately reflect routine operational performance, whereas long-term IQC data capture the cumulative effects of day-to-day laboratory practice, reagent changes, calibration cycles, and environmental factors [8].

Despite the routine availability of IQC data in most clinical laboratories, there are limited published studies demonstrating the systematic use of long-term, routine IQC-derived analytical imprecision for RCV estimation, particularly within primary healthcare laboratory networks [9]. This gap is more pronounced in large, decentralized systems where multiple analyzers operate under harmonized protocols, yet local operational variability may still influence analytical performance. Evaluating IQC-derived CV_a_ across such networks provides an opportunity to generate analytically realistic RCVs that are directly applicable to routine clinical interpretation [6].

In primary healthcare, laboratories play a central role in the longitudinal monitoring of biochemical markers used in the management of non-communicable diseases (NCD), including diabetes mellitus, dyslipidemia, and chronic kidney disease [10]. These conditions require repeated testing over extended periods, and patient results often fluctuate near clinical decision limits [11]. In this context, inappropriate interpretation of analytically insignificant changes may lead to unnecessary follow-up investigations or unwarranted modification of therapy [12]. Application of RCV derived from routine analytical performance offers a pragmatic approach to support more consistent and evidence-based interpretation of serial results in NCD monitoring [13]. While RCVs have been widely described, most published estimates are derived from controlled validation experiments or single-laboratory datasets. Data reflecting long-term routine analytical performance across decentralized primary healthcare laboratory networks remains limited. Such environments may experience additional operational variability related to reagent lot changes, calibration cycles, and workflow differences [14].

The present study therefore evaluates long-term CV_a_ across a harmonized network of Malaysian primary healthcare laboratories using routinely generated IQC data. By integrating these real-world performance estimates with biological variation data, this study aims to derive analytically realistic RCV applicable to longitudinal monitoring of NCD biomarkers in primary care settings.

## 2. Materials and Methods

### 2.1. Study Design and Setting

This was a retrospective, observational analytical performance study based on routinely generated IQC data collected from government primary healthcare laboratories (Klinik Kesihatan, KK) in Malaysia. The study evaluated long-term CV_a_ and its implications for RCV application in longitudinal monitoring of selected NCD biomarkers under real-world primary care operating conditions.

### 2.2. Participating Laboratories and Analytical Systems

This study was conducted across 29 government primary healthcare laboratories in Malaysia. A total of 32 Beckman Coulter AU480 automated chemistry analyzers were included; in instances where a laboratory operated two units, each was treated as an independent analytical identifier (KK01–KK32) for data analysis. All analyses utilized commercially available Medicon reagents on the AU480 platform, following manufacturer instructions and established laboratory operating procedures. Assays were confirmed to be traceable to higher-order reference systems.

### 2.3. Analytes Included

Six routine biochemical analytes were included: glucose, creatinine, total cholesterol (Chol), triglycerides, high-density lipoprotein (HDL) cholesterol, and alanine aminotransferase (ALT).

### 2.4. IQC Data Collection

IQC data were collected over a 22-month observation window between January 2024 and October 2025. For each participating laboratory, a continuous 22-month dataset with two concentration levels (Level 1 and Level 2) for each analyte was extracted for analysis to ensure comparable evaluation of long-term analytical imprecision across sites. For each analytical unit and IQC level, the number of IQC measurements (*n*) and the coefficient of variation (CV, %) were recorded. Prior to the study, all sites completed local method verification according to CLSI EP15-A2 guidelines to ensure observed precision met manufacturer claims [15]. 

All participating laboratories followed routine internal quality control procedures in accordance with the Ministry of Health (MOH) laboratory quality management requirements [16]. IQC results that violated Westgard control rules were investigated and resolved through routine troubleshooting procedures before subsequent analytical runs. Therefore, the IQC data included in this study represent validated routine operational performance after standard quality control management [17].

### 2.5. Estimation of CV_a_

CV_a_ was estimated using routinely recorded IQC CV values and calculated separately for Level 1 and Level 2 QC materials to account for potential concentration-dependent analytical variability. For each analyte and QC level, the median CV was used to represent typical analytical performance, while the 90th percentile (P90) CV was used to characterize upper-bound imprecision. The use of median and percentile-based estimates reduces sensitivity to outliers and reflects stable long-term performance rather than short-term fluctuations.

### 2.6. RCV Calculation

RCVs were derived to define the minimum difference between two consecutive results from the same individual likely to represent true physiological change. The standard symmetrical RCV formula was used

RCV (%) = Z × √2 × √(CV_a_^2^ + CV_i_^2^) (1)

A Z-score of 1.96 was applied, representing a two-sided probability of 95% (significant change). Symmetrical RCV was used in this study, assuming equal probability of increase or decrease in analyte values; asymmetric RCV was not evaluated. CV_a_ was derived from the median observed routine IQC CV values at both concentration levels to reflect “real-world” operating conditions. CV_i_ is a within-subject biological variation estimate obtained from the European Federation of Clinical Chemistry and Laboratory Medicine (EFLM) Biological Variation Database [18].

### 2.7. Statistical Analysis

Descriptive statistics were performed using R (version 4.5.0). The median was used to represent typical analytical performance, while the P90 served as a robust indicator of upper-bound imprecision across the 32 units. QC implementation efficiency was assessed by analyzing the distribution and mode of QC testing frequency (*n*). All data were stratified by analyte and QC level to ensure a comprehensive characterization across clinically relevant measurement ranges.

## 3. Results

### 3.1. Quality Control Practice and Testing Frequency (n)

In routine Malaysian primary healthcare, IQC is typically expected once per working day, totaling approximately 20–23 measurements per month. In this study, the frequency of IQC testing (n) was consistently 20 measurements per month across all analytes for both Level 1 and Level 2. The mode for most analytes was 19, 20, or 21, which indicates a highly standardized practice of running IQC roughly once per working day. Upper-bound performance of P90 values for IQC frequency ranged from 23.0 to 24.7 measurements per month, confirming that most laboratories adhered strictly to the upper range of expected daily testing. Table 1 summarizes the testing frequency for all analytes, while the corresponding frequency distributions across the laboratories are illustrated as histograms in Appendix A.1. Overall, the observed IQC frequency distributions indicate that IQC testing for the evaluated analytes was implemented in a regular and consistent manner across participating primary healthcare laboratories, providing a stable operational basis for subsequent evaluation of analytical imprecision and longitudinal result interpretation.

### 3.2. Analytical Imprecision

Glucose, total cholesterol, and triglycerides demonstrated the lowest within-laboratory CV_a_, with median values consistently clustered between 1.7% and 1.9% at both IQC concentration levels. HDL cholesterol exhibited slightly higher median imprecision, with CV_a_ values of approximately 2.5–2.6%, but remained comparatively stable across concentration levels.

A clear concentration-dependent pattern was observed for ALT and creatinine, with higher median CV_a_ values at the L1 compared to L2. Specifically, median CV_a_ values for ALT decreased from 3.79% (L1) to 2.60% (L2), while creatinine showed a reduction from 3.52% (L1) to 2.69% (L2). This trend suggests increased analytical variability at lower analyte concentrations, likely reflecting reduced signal-to-noise ratios or greater susceptibility to methodological variation.

**Figure A14:**
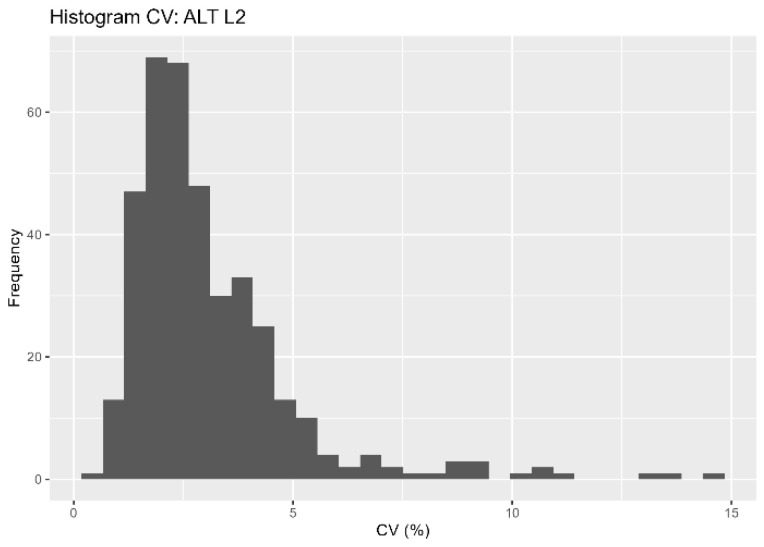
Distribution of Analytical Imprecision (CV%) for ALT at QC Level 2.

The P90 CV_a_ values, representing the upper bound of routine CV_a_ across participating laboratories, ranged from 3.19% for glucose (L1) to 6.14% for creatinine (L1). These findings indicate that the majority of laboratories maintained acceptable CV_a_ under routine operating conditions. A summary of median and P90 CV_a_ values for all analytes is provided in Table 2, while detailed frequency distributions are presented as histograms in Appendix A Figure A14.

**Figure 1:**
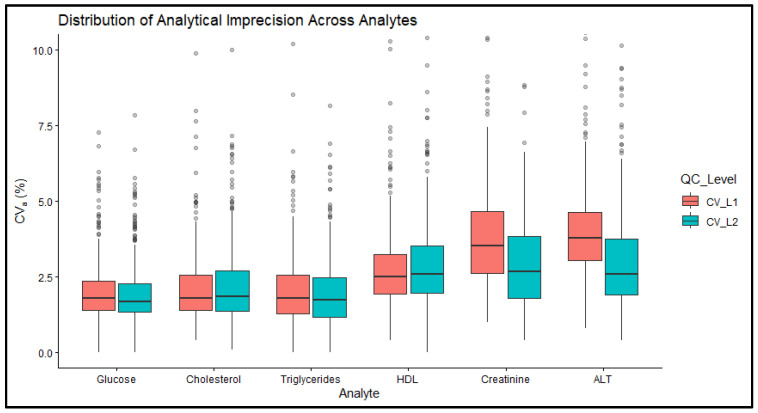
Distribution of CV_a_ across analytes and IQC levels. Boxes represent the interquartile range with median lines; whiskers indicate the range excluding outliers.

A clearer visualization of CV_a_ distributions across analytes and IQC levels is shown in Figure 1. Median CV_a_ values for glucose, total cholesterol, and triglycerides were consistently maintained below 2.5% at both IQC levels. In contrast, HDL cholesterol, creatinine, and ALT displayed higher median CV_a_ values and wider interquartile ranges, with ALT showing the highest median imprecision at approximately 4.0% for the L1 control. Although most laboratories demonstrated stable analytical performance, outliers exceeding 7.5% CV_a_ were observed across all analytes, particularly for creatinine and ALT.

### 3.3. Derived Reference Change Values and Sensitivity Analysis

Analyte-specific RCVs were derived by integrating the observed median CV_a_ with established CV_i_. RCVs were calculated separately at Level 1 and Level 2 IQC concentrations to account for concentration-dependent analytical imprecision and to reflect clinically relevant monitoring ranges. Meanwhile, sensitivity analysis is performed using the upper-bound P90 CV_a_ to evaluate the effect of higher CV_a_ on the derived RCV.

Table 3 shows the derived RCV and sensitivity analysis for all six analytes. Triglycerides exhibited the highest RCV at 55.1%, driven by their high CV_i_ of 19.8%. Cholesterol and HDL-cholesterol showed lower RCV, ranging from 15.8% to 17.4%. Glucose and creatinine demonstrated relatively low RCVs of approximately 14% and 14–16%, respectively, suggesting that small percentage shifts in these analytes are more likely to represent true physiological changes. Sensitivity analysis shows that the largest differences were observed for creatinine and ALT, reflecting their relatively higher analytical imprecision. In contrast, triglycerides showed minimal change in RCVs despite the use of higher CV_a_, consistent with its large CV_i_, which dominates the overall RCV calculation. Overall, the relative ranking of analytes and the general interpretation of RCV thresholds remained unchanged, indicating that the derived RCV estimates are robust to P90 CV_a_.

### 3.4. Clinical Application of the RCV

**Figure 2:**
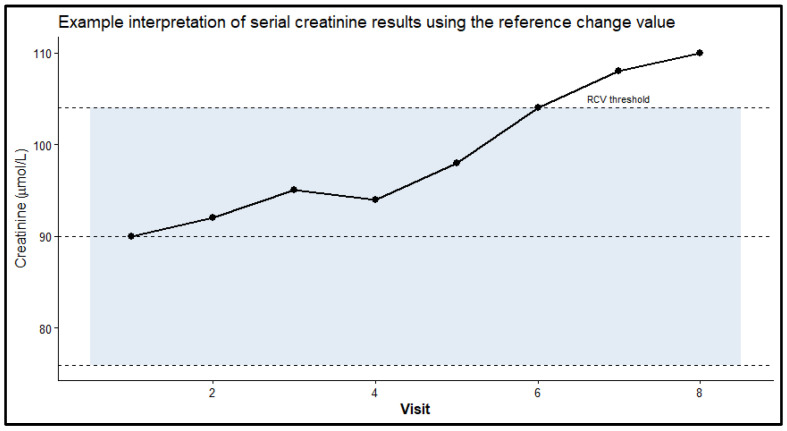
Clinical interpretation of serial creatinine results using the derived RCV. The shaded region represents the interval of expected analytical and within-subject biological variation around the baseline result. Serial values exceeding this interval indicate statistically significant change beyond expected routine variability.

To demonstrate the clinical utility of these variations, Figure 2 illustrates the interpretation of serial creatinine results using the derived RCV. The shaded region defines the interval of expected combined analytical and biological variation around a baseline measurement. As shown in the example, serial values remaining within this shaded region represent routine variability, whereas values exceeding the upper RCV threshold (observed from Visit 6 onwards) indicate a statistically significant clinical change.

### 3.5. Comparison of Analytical and Biological Variation

**Figure 3:**
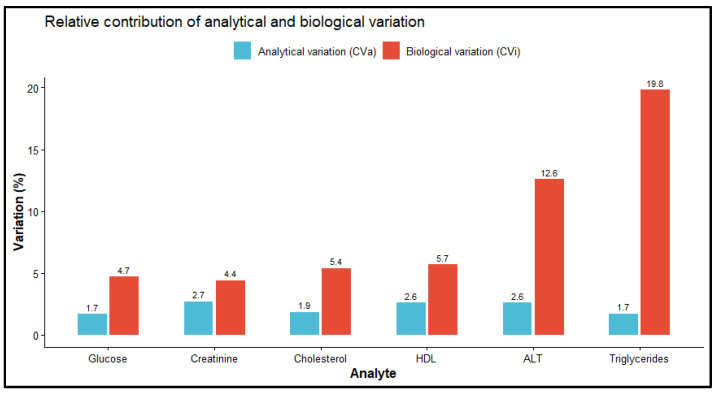
Relative contributions of CV_a_ and CV_i_ for the six biochemical analytes evaluated. Bars represent median analytical imprecision derived from IQC data and published CV_i_ estimates.

The relative contributions of CV_a_ and CV_i_ for the six evaluated analytes are illustrated in Figure 3. For all biochemical markers, CV_i_ was identified as the primary contributor to total variation, significantly exceeding analytical imprecision. The most pronounced difference was observed for Triglycerides, where CV_i_ (19.8%) was more than ten-fold higher than the observed CV_a_ (1.7%). Conversely, creatinine showed the narrowest gap between variables (CV_a_: 2.7%; CV_i_: 4.4%). These results indicate that while analytical performance is well controlled, clinical interpretation must heavily account for inherent biological fluctuations.

## 4. Discussion

The results of this study provide a real-world characterization of analytical performance across a harmonized network of 32 analytical units in Malaysian primary healthcare laboratories. By utilizing routine 22-month IQC data, we have derived RCV that reflects actual operational conditions rather than the idealized, controlled settings typically provided by manufacturers. The novelty of this study lies in its shift away from traditional single-center or short-term evaluations; instead, it leverages extensive longitudinal data across a decentralized network to provide analytically realistic RCVs reflective of daily clinical practice. This multi-center approach captures the inherent inter-laboratory variability of a large-scale primary care framework while maintaining a sufficient data duration per site for a reliable estimation of CV_a_. Consequently, our findings offer a more representative basis for the longitudinal interpretation of patient results than conventional studies, providing clinicians with benchmarks directly applicable to routine primary healthcare settings.

The foundation of any robust RCV is a stable and consistent IQC process [19]. Our analysis of QC testing frequency demonstrated highly standardized practices across the 29 participating laboratories, with a consistent median of 20 measurements per month for both concentration levels. This standardized approach aligns with recommendations by Thelen et al. [20], who emphasize that long-term IQC data capture the cumulative effects of routine operational factors such as reagent lot changes, calibration cycles, and day-to-day workflow variability. Adherence to a “once per working day” protocol confirms that these units operate under a stable quality management framework, ensuring that imprecision estimates are based on a reliable and representative dataset.

A key observation in this study was the concentration-dependent nature of CV_a_, particularly for ALT and creatinine. For creatinine, the median analytical imprecision CV_a_ decreased from 3.52% at Level 1 to 2.69% at Level 2. This finding supports the argument by Panteghini and Sandberg [21] that residual imprecision persists even when assays meet regulatory requirements, potentially influencing the interpretation of small changes between consecutive results. Such higher imprecision at lower concentrations is clinically significant in primary care, where the early detection of chronic kidney disease often relies on subtle shifts in creatinine levels near the lower limit of the reference range [22]. By utilizing the median alongside the P90 as an upper-bound indicator, we provide a pragmatic assessment that accounts for routine variability while remaining robust against transient outliers. The median was used to represent typical analytical performance where data may not follow a normal distribution and may include occasional extreme values. The P90 was selected as a pragmatic estimate of upper-bound routine imprecision, representing the level below which 90% of observed CV_a_ values fall. Alternative metrics such as mean ± standard deviation assume normal distribution and are sensitive to outliers, while maximum values or higher percentiles (e.g., P95) may overemphasize rare extreme observations. Conversely, lower percentiles (e.g., Q3) may underestimate upper-bound variability. This approach is consistent with prior studies utilizing large-scale IQC datasets and emphasizing robust evaluation of analytical performance under routine conditions, where variability across laboratories necessitates the use of percentile-based descriptors rather than parametric assumptions [3,20]. Therefore, P90 provides a balanced and robust representation of higher-end routine imprecision, capturing inter-laboratory variability while minimizing distortion from extreme values.

By integrating observed “real-world” CV_a_ with contemporary CV_i_ from the EFLM database, we derived RCV directly applicable to routine clinical practice [7]. Our findings confirm that CV_i_ is the dominant contributor to RCV magnitude for most analytes. This aligns with the work of Aarsand et al. [23], who highlight that understanding the interplay between CV_a_ and CV_i_ is essential to avoid the overinterpretation of small numerical differences. For instance, the high RCV for triglycerides (55.1%) indicates that a change must exceed 50% to be statistically significant, whereas analytes with lower biological variability, such as glucose and creatinine, yielded much tighter RCVs of approximately 14% and 14–16%, respectively. These differences underscore the limitations of applying a uniform percentage change threshold across different biochemical markers.

The RCVs derived in the present study were generally comparable with those reported in previous investigations. For example, Bugdayci et al. [24] reported RCV of 14.63% for glucose, 15.03% for creatinine, and 46.62% for triglycerides using routine clinical chemistry analyzer data. Similarly, Hong et al. [25] reported RCV of 22.00% for glucose and 27.44% for creatinine when applying RCV-based delta check optimization. The estimates obtained in this study fall within similar ranges while reflecting the performance observed across multiple decentralized primary healthcare sites. These findings suggest that RCV derived from routine IQC data is consistent with established literature while offering greater local relevance [24,25]. The derived RCV therefore provides practical thresholds for interpreting serial laboratory results in clinical practice. Changes exceeding the RCV are unlikely to be explained by expected analytical and biological variation alone and may indicate true physiological change [26]. As illustrated in Figure 2, applying RCV thresholds to serial creatinine measurements provides a simple framework for distinguishing meaningful changes from routine variability. Such interpretation may assist clinicians in monitoring disease progression, assessing therapeutic response, or detecting early deterioration in patient status.

The clinical utility of these RCVs is most evident in the longitudinal monitoring of NCD, where results often fluctuate near clinical decision limits [7]. Previous studies have demonstrated that clinicians often rely on subjective judgment when interpreting changes in consecutive results, leading to variability in assessing clinical significance [6,27,28]. This highlights the importance of objective statistical tools to support standardized interpretation. In diabetes and renal health management, the relatively low RCV for glucose and creatinine enables clinicians to more accurately detect significant trends indicating disease progression [29]. Conversely, for dyslipidemia, understanding that a 55% change is required for triglycerides prevents the overinterpretation of minor fluctuations, thereby minimizing unnecessary repeat testing and patient anxiety [30]. Integrating these RCV concepts into laboratory reporting may ultimately promote more informed and standardized longitudinal patient care in primary healthcare settings.

Several limitations of this study should be acknowledged. First, CV_a_ was estimated using routine IQC data rather than replicate patient sample measurements, which may not fully capture all sources of analytical variation encountered in clinical specimens. Second, the analysis was limited to laboratories operating a single analytical platform, which may restrict the generalizability of the derived RCVs to other analytical systems. Third, CV_i_ estimates were obtained from the EFLM Biological Variation Database and may not fully represent biological variability within the Malaysian population. CV_i_ values used in this study were obtained from the EFLM Biological Variation Database, which represents the most standardized and internationally accepted reference for biological variation. We acknowledge that these values may not fully reflect population-specific biological variation within the Malaysian setting. However, the present study was designed as a first-phase analysis, with the primary objective of establishing robust and stable estimates of CV_a_ across a harmonized primary healthcare laboratory network. The use of EFLM CV_i_ in this phase ensures methodological consistency and enables comparability with existing literature while focusing on validating the analytical component of the RCV framework. Subsequent phases of this work will involve the evaluation and integration of locally derived CV_i_ estimates based on longitudinal patient data from the same healthcare setting. This stepwise approach ensures that population-specific adjustments are built upon a well-characterized and stable analytical foundation, which is the focus and novelty of the current study.

Despite these limitations, the use of long-term IQC data across a large primary healthcare laboratory network provides a realistic representation of routine analytical performance and offers a pragmatic framework for deriving RCV applicable to real-world clinical practice.

## 5. Conclusions

In conclusion, this study provides analytically derived RCV for six commonly measured biochemical analytes based on long-term IQC data obtained under routine laboratory conditions. The analyte-specific RCV derived in this study supports more accurate interpretation of serial biochemical results and offers a practical tool for improving longitudinal monitoring of NCD by distinguishing true physiological change from expected analytical and biological variability.

## Figures and Tables

**Table 1:** Monthly frequency of IQC measurements across six biochemical analytes in primary healthcare laboratories.

Analyte	Level	*n*	Median	P90	Mode
ALT	L1	384	20	24.0	20
ALT	L2	384	20	24.0	20
Chol	L1	384	20	24.0	19
Chol	L2	384	20	24.0	20
Creatinine	L1	384	20	24.7	21
Creatinine	L2	384	20	24.7	21
Glucose	L1	373	20	24.0	20
Glucose	L2	373	20	24.0	20
HDL	L1	383	20	24.0	21
HDL	L2	383	20	24.0	20
Triglycerides	L1	383	20	23.0	21
Triglycerides	L2	383	20	23.0	19

Abbreviations: Chol, total cholesterol; HDL, high-density lipoprotein cholesterol; ALT, alanine aminotransferase; L1, level 1; L2, level 2; *n*, total number of IQC measurements.

**Table 2:** Distribution of within-laboratory analytical imprecision (CV%) for six biochemical analytes in primary healthcare laboratories.

Analyte	Level	*n*	Median	P90
ALT	CV_L1	384	3.790	5.803
ALT	CV_L2	384	2.595	4.938
Chol	CV_L1	384	1.790	3.507
Chol	CV_L2	384	1.860	3.694
Creatinine	CV_L1	384	3.515	6.138
Creatinine	CV_L2	384	2.685	4.884
Glucose	CV_L1	373	1.810	3.188
Glucose	CV_L2	373	1.670	3.334
HDL	CV_L1	383	2.500	4.144
HDL	CV_L2	383	2.600	4.688
Triglycerides	CV_L1	383	1.800	3.328
Triglycerides	CV_L2	383	1.730	3.414

Abbreviations: Chol, total cholesterol; HDL, high-density lipoprotein cholesterol; ALT, alanine aminotransferase; L1, level 1; L2, level 2; *n*, total number of IQC measurements.

**Table 3:** Derived reference change values and sensitivity analysis.

		Level 1				Level 2			
Analytes	CV_i_ (%)	CV_a_ Median	CV_a_ P90	RCV Median	RCV P90	CV_a_ Median	CV_a_ P90	RCV Median	RCV P90
ALT	12.6	3.79	5.80	36.5	38.5	2.60	4.94	35.7	37.5
Chol	5.4	1.79	3.51	15.8	17.8	1.86	3.69	15.8	18.1
Creatinine	4.4	3.52	6.14	15.6	20.9	2.68	4.88	14.3	18.2
Glucose	4.7	1.81	3.19	14.0	15.7	1.67	3.33	13.8	16.0
HDL	5.7	2.50	4.14	17.3	19.5	2.60	4.69	17.4	20.5
Triglycerides	19.8	1.80	3.33	55.1	55.7	1.73	3.41	55.1	55.7

Abbreviations: Chol, total cholesterol; HDL, high-density lipoprotein cholesterol; ALT, alanine aminotransferase. Note: RCV (%) = 1.96 × √2 × √(CV_a_^2^ + CV_i_^2^). Median CV_a_ represents typical routine analytical imprecision; P90 CV_a_ represents upper-bound imprecision. RCV were expressed as percentage change; absolute change thresholds can be derived for a given baseline result by multiplying the baseline concentration by the corresponding RCV (%). CV_i_ value is derived from the EFLM database.

**Figure A1:**
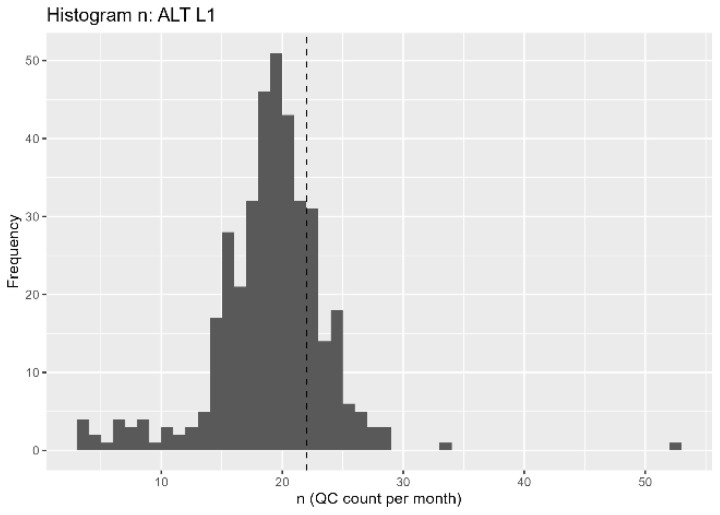
Distribution of Monthly Quality Control Testing Fre-quency for ALT at Level 1.

**Figure A2:**
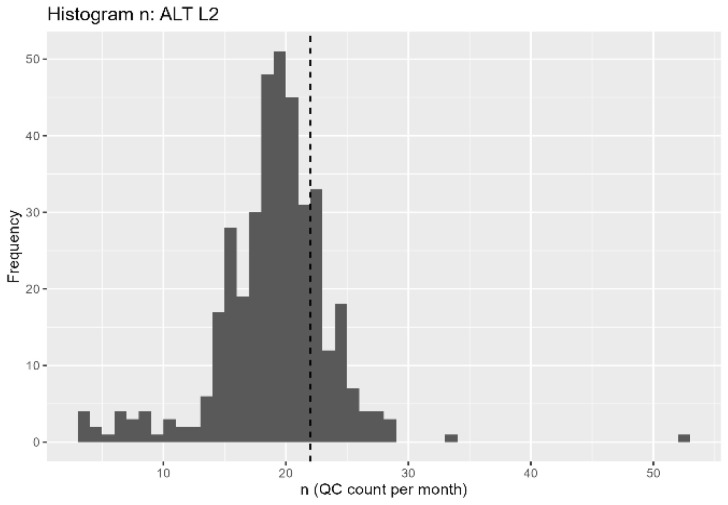
Distribution of Monthly Quality Control Testing Frequency for ALT at Level 2.

**Figure A3:**
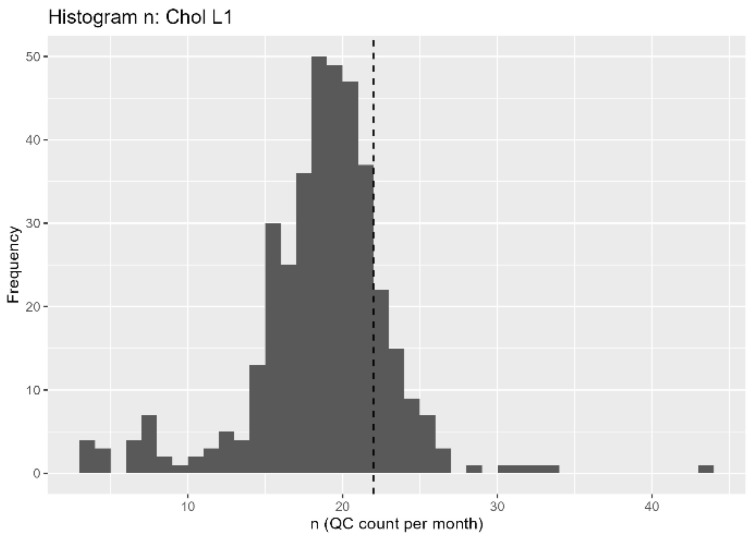
Distribution of Monthly Quality Control Testing Frequency for Chol at Level 1.

**Figure A4:**
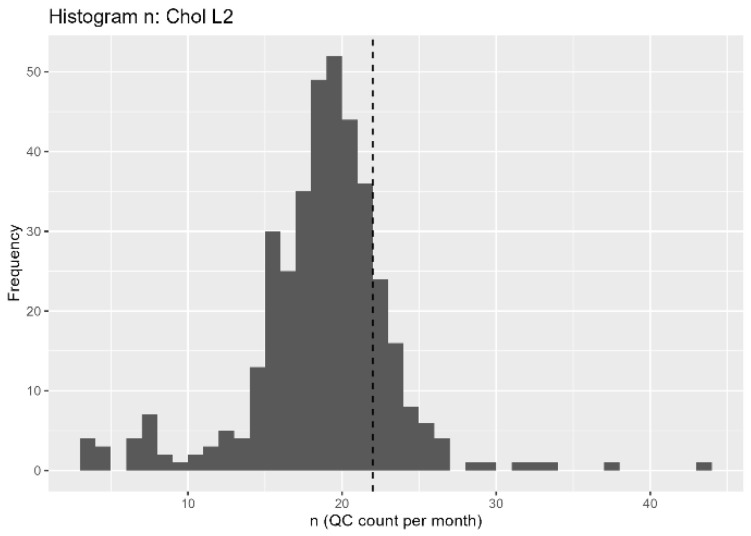
Distribution of Monthly Quality Control Testing Frequency for Chol at Level 2.

**Figure A5:**
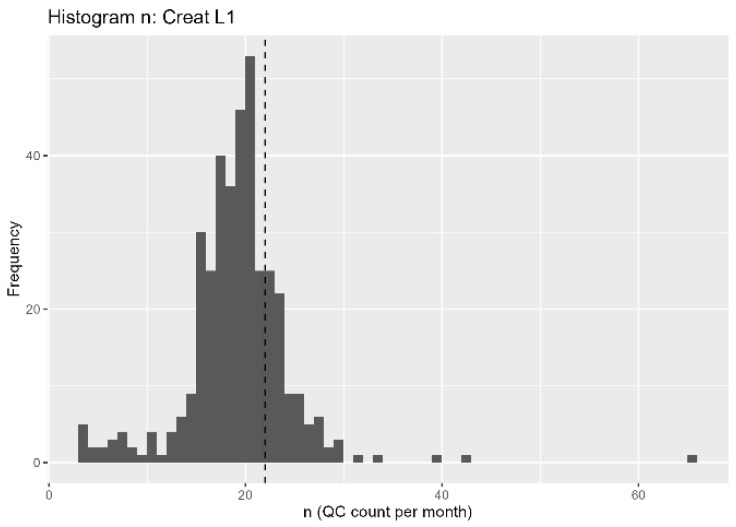
Distribution of Monthly Quality Control Testing Frequency for Creatinine at Level 1.

**Figure A6:**
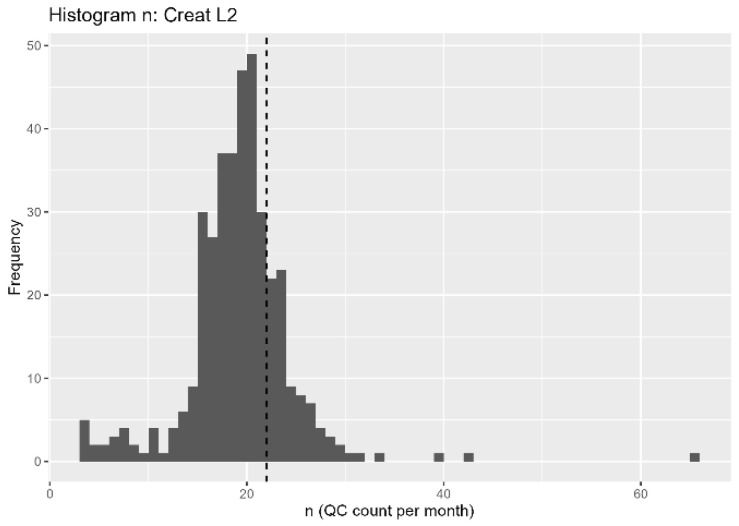
Distribution of Monthly Quality Control Testing Frequency for Creatinine at Level 2.

**Figure A7:**
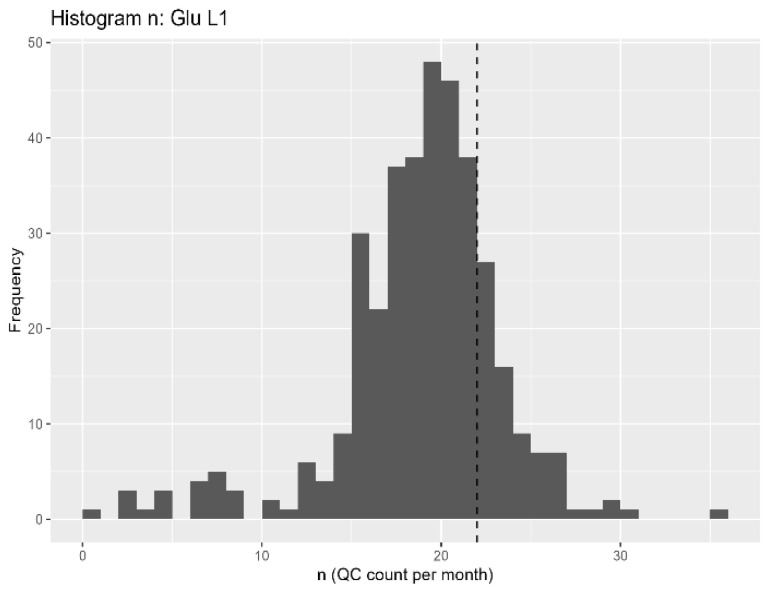
Distribution of Monthly Quality Control Testing Frequency for Glucose at Level 1.

**Figure A8:**
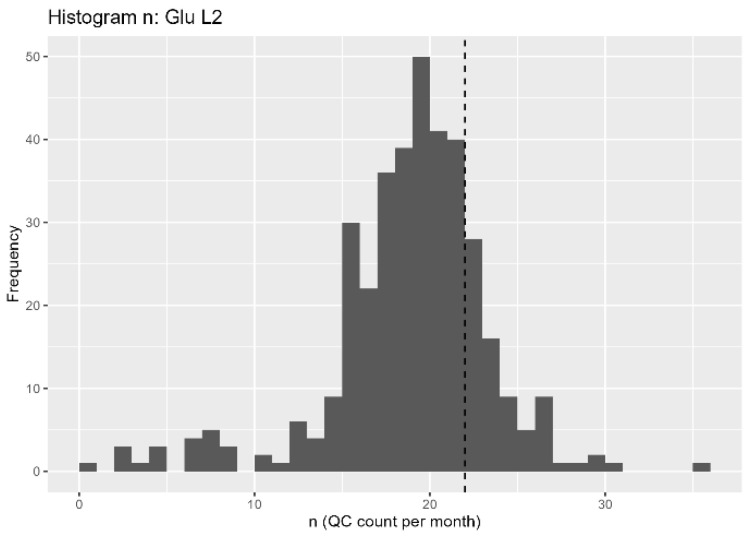
Distribution of Monthly Quality Control Testing Frequency for Glucose at Level 2.

**Figure A9:**
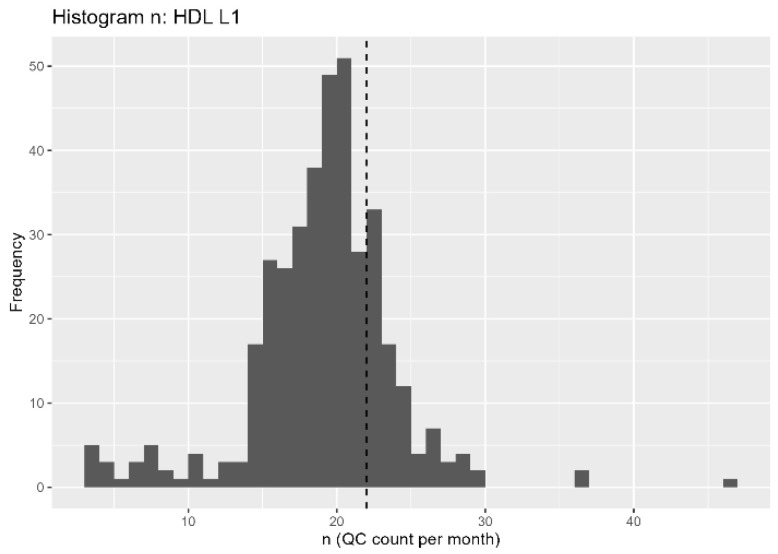
Distribution of Monthly Quality Control Testing Frequency for HDL at Level 1.

**Figure A10:**
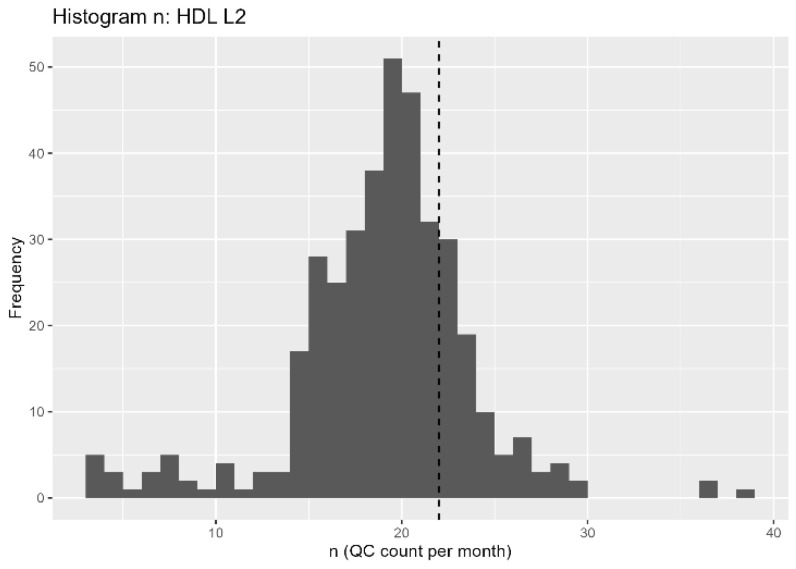
Distribution of Monthly Quality Control Testing Frequency for HDL at Level 2.

**Figure A11:**
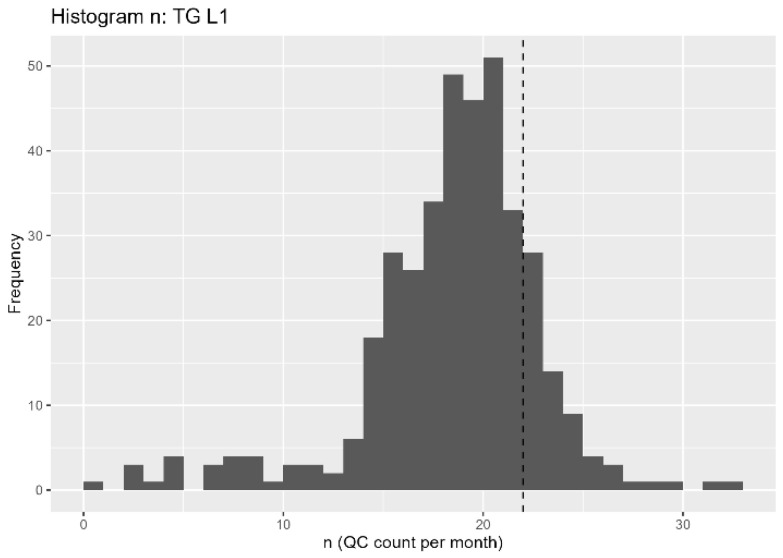
Distribution of Monthly Quality Control Testing Frequency for Triglycerides at Level 1.

**Figure A12:**
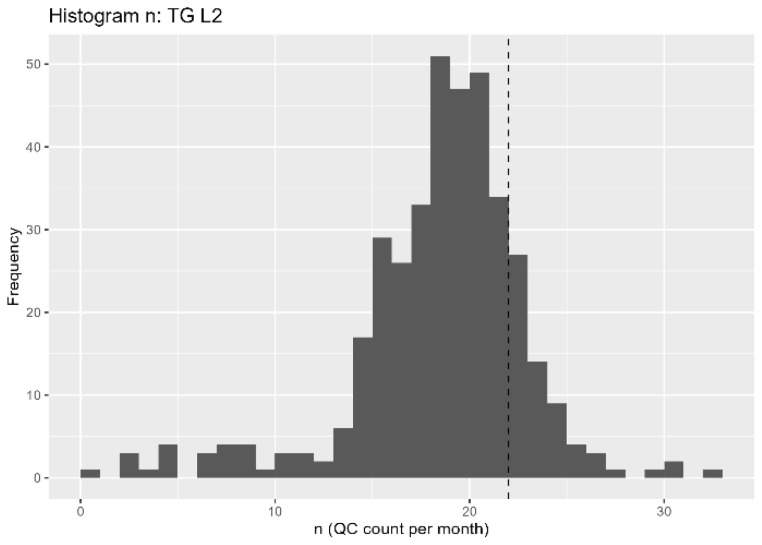
Distribution of Monthly Quality Control Testing Frequency for Triglycerides at Level 2.

**Figure A13:**
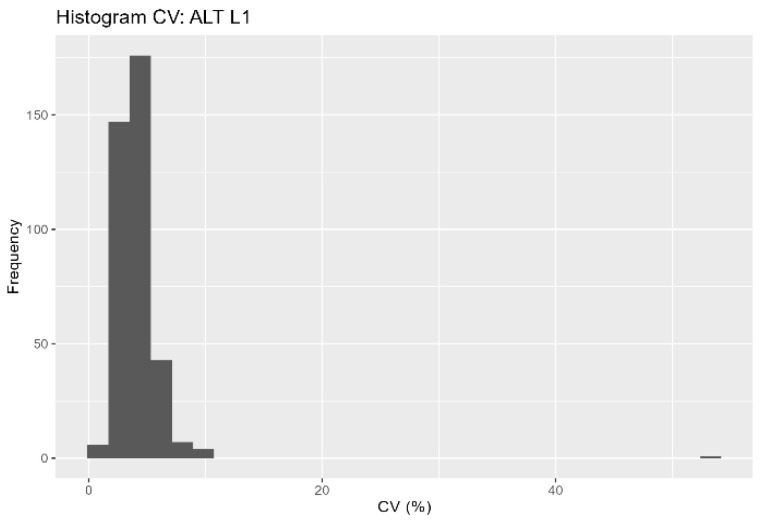
Distribution of Analytical Imprecision (CV%) for ALT at QC Level 1.

**Figure A15:**
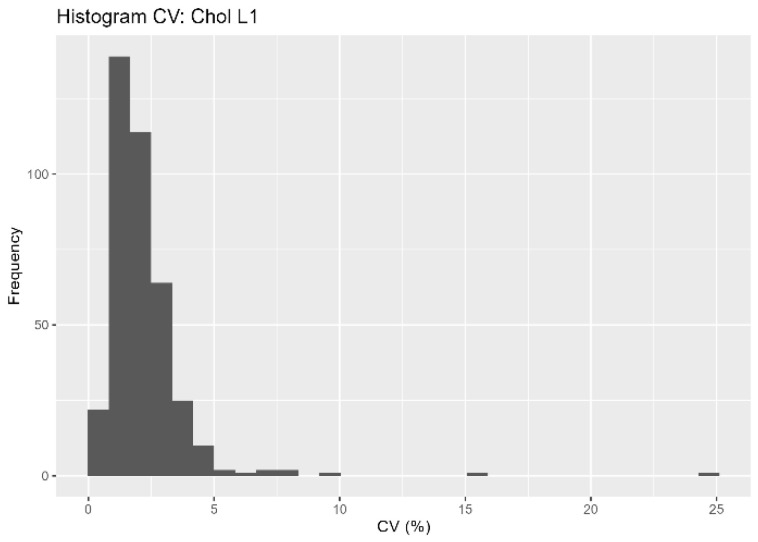
Distribution of Analytical Imprecision (CV%) for Chol at QC Level 1.

**Figure A16:**
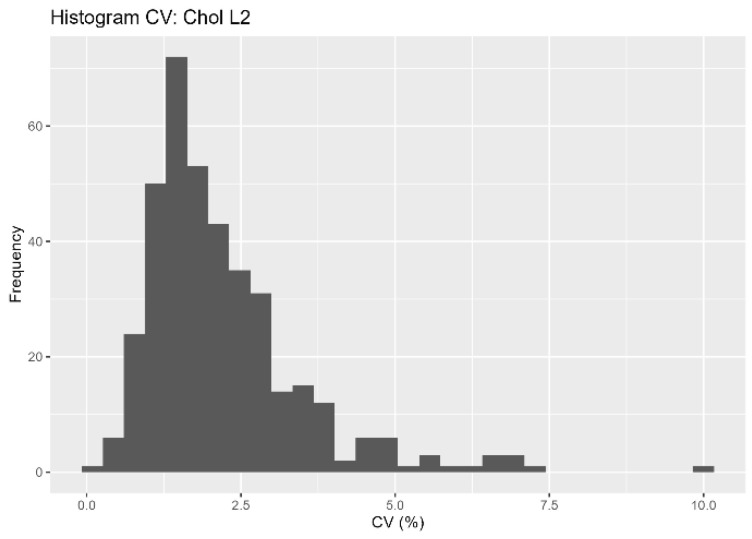
Distribution of Analytical Imprecision (CV%) for Chol at QC Level 1.

**Figure A17:**
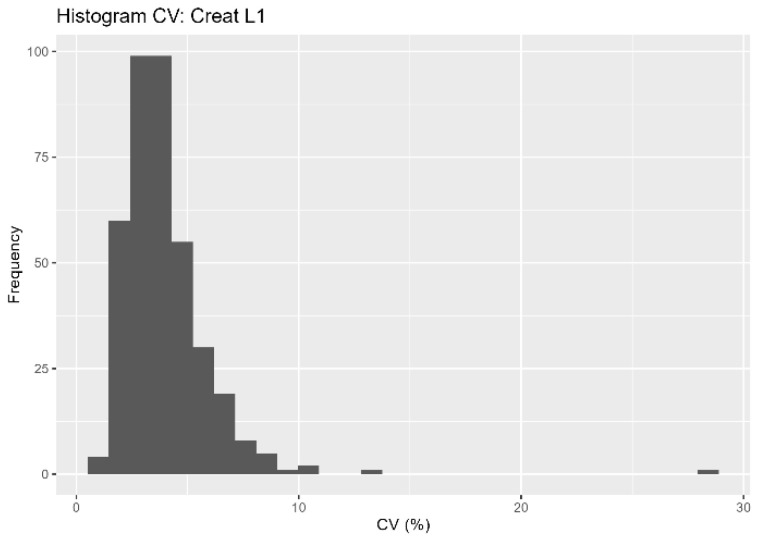
Distribution of Analytical Imprecision (CV%) for Creatinine at QC Level 1.

**Figure A18:**
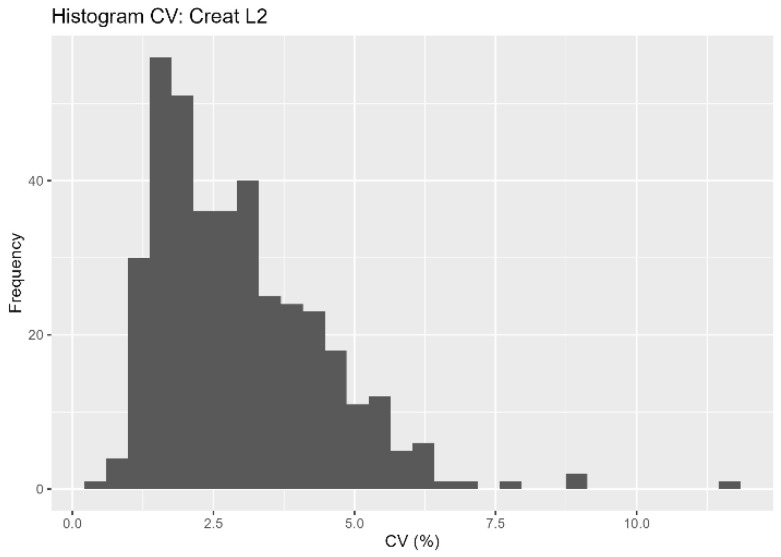
Distribution of Analytical Imprecision (CV%) for Creatinine at QC Level 2.

**Figure A19:**
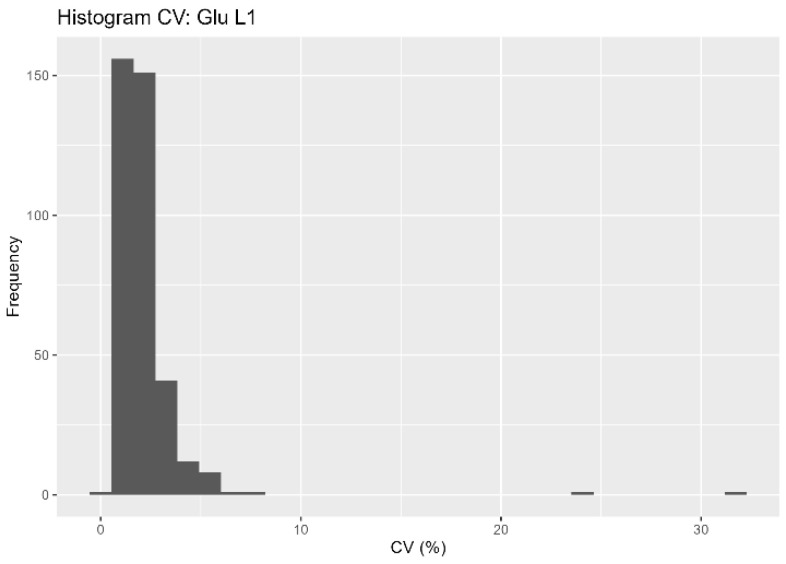
Distribution of Analytical Imprecision (CV%) for Glucose at QC Level 1.

**Figure A20:**
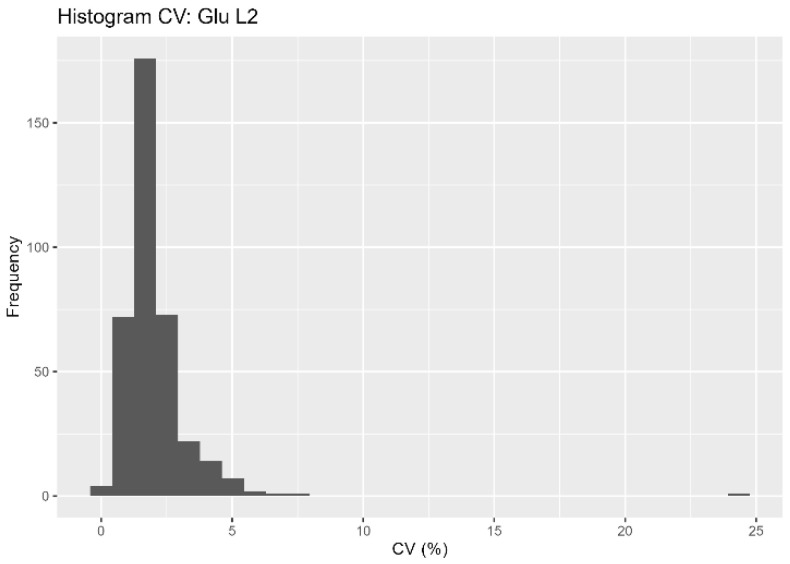
Distribution of Analytical Imprecision (CV%) for Glucose at QC Level 2.

**Figure A21:**
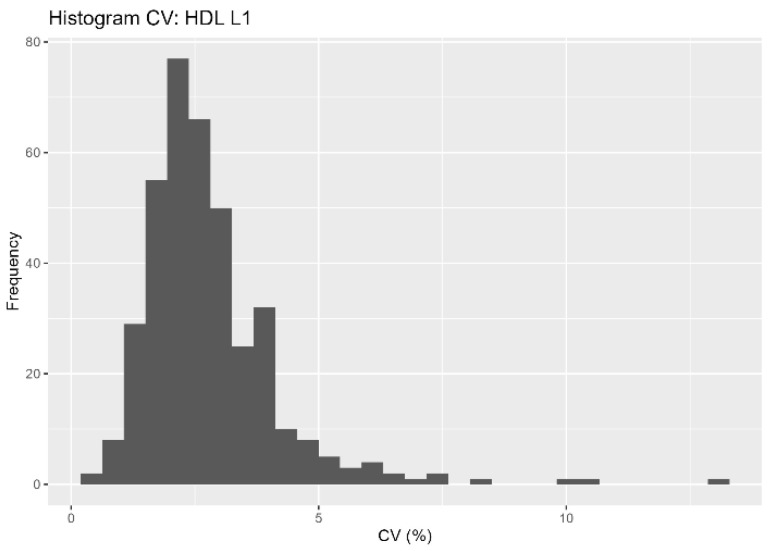
Distribution of Analytical Imprecision (CV%) for HDL at QC Level 1.

**Figure A22:**
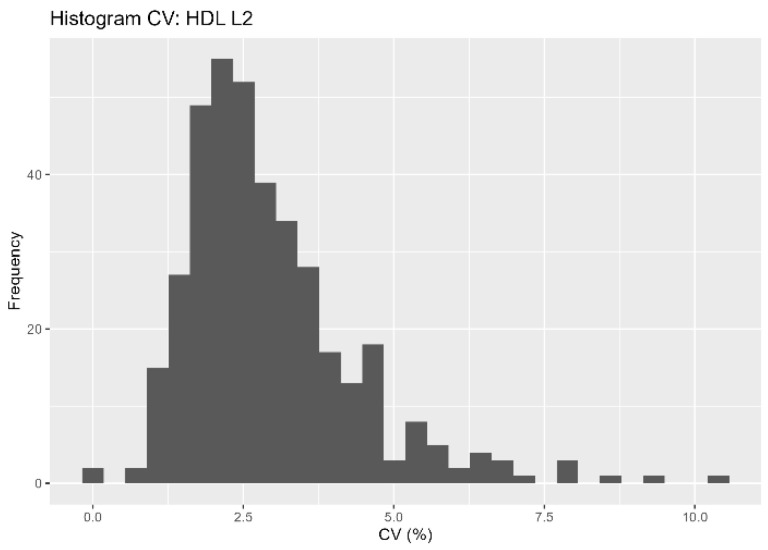
Distribution of Analytical Imprecision (CV%) for HDL at QC Level 2.

**Figure A23:**
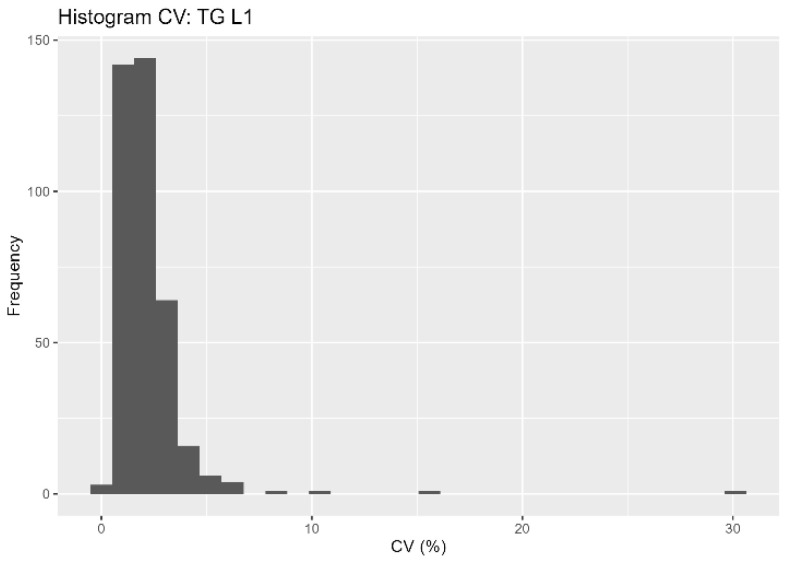
Distribution of Analytical Imprecision (CV%) for Triglycerides at QC Level 1.

**Figure A24:**
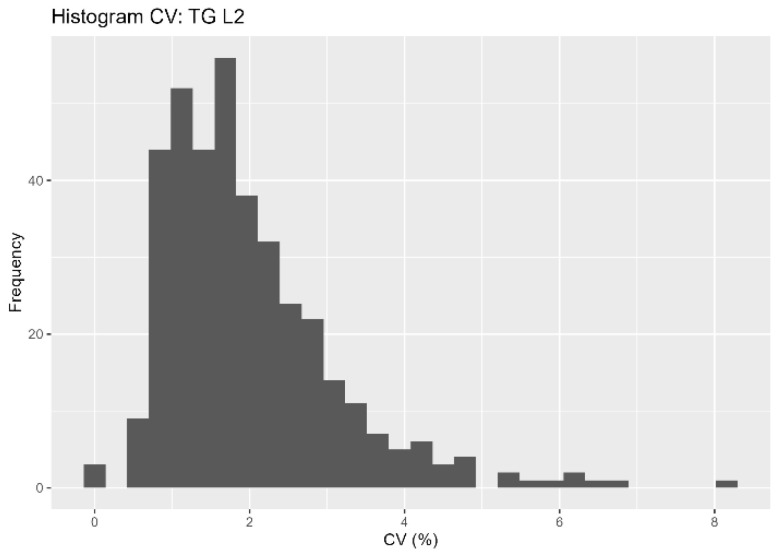
Distribution of Analytical Imprecision (CV%) for Triglycerides at QC Level 1.

## Data Availability

The original contributions presented in this study are included in the article and Appendix A. Further inquiries can be directed to the corresponding authors.

## Abbreviations

The following abbreviations are used in this manuscript:

ALT	Multidisciplinary Digital Publishing Institute
CV_a_	Analytical imprecision
CV_i_	Biological variation
CV	Coefficient of variation
Chol	Cholesterol
EFLM	European Federation of Clinical Chemistry and Laboratory Medicine
HDL	High-density lipoprotein cholesterol
IQC	Internal quality control
KK	Klinik Kesihatan
L1	Level 1
L2	Level 2
MOH	Ministry of Health
NCD	Non-communicable disease
P90	Percentile 90
